# Tracheobronchial metastasis from atypical carcinoid

**DOI:** 10.36416/1806-3756/e20250267

**Published:** 2025-11-14

**Authors:** Alan Jhunior Solis, Jimmy Icaza-Vera, Javier Flandes

**Affiliations:** 1. Unidad de Broncoscopia y Neumología Intervencionista, Hospital Universitario Fundación Jiménez Díaz, Madrid, España.

**Figure 1:**
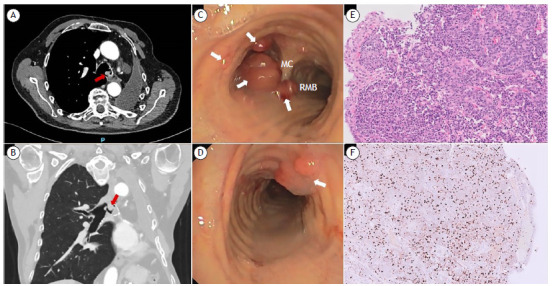
In A, axial CT scan showing a nodular endobronchial lesion with soft tissue density (red arrow). In B, frontal CT scan showing the same nodular lesion (diameter, 19 mm; red arrow). In C and D, flexible bronchoscopy showing polypoid lesions at the entrance of the left main bronchus, main carina, and trachea (white arrows). In E, pathological examination showing well-differentiated neuroendocrine cells forming nests and trabeculae (H&E; magnification, ×10). In F, immunohistochemistry showing a Ki-67 proliferation index of 50%. MC: main carina; and RMB: right main bronchus.

A 78-year-old male with no relevant medical history was diagnosed with atypical carcinoid in 2012 and underwent left upper lobectomy with curative intent. Three years later, the patient presented with a single local recurrence, and a left pneumonectomy was performed. Twelve years after the initial diagnosis, a CT scan was performed because of dyspnea, revealing multiple round lesions in the tracheobronchial tree (Figures 1A and 1B). Bronchoscopy showed several polypoid lesions (Figures 1C and 1D), and cryobiopsies were performed. Pathological examination showed well-differentiated neuroendocrine cells, and immunohistochemistry showed a Ki-67 proliferation index of 50%, as well as positivity for CD56, synaptophysin, and chromogranin A, the final diagnosis being atypical carcinoid (Figures 1E and 1F). During a second bronchoscopy, laser and electrocautery resection of the tracheobronchial lesions was performed. 

The most common site of carcinoid tumors is the gastrointestinal tract, followed by the tracheobronchial tree.^1^ Diagnosis requires a biopsy with histological confirmation, and atypical carcinoids are less common than typical carcinoids, the recurrence rate for the former being higher than that for the latter.^1^ ^,^ ^2^ Atypical carcinoid usually presents as a peripheral lung lesion or a solitary endobronchial lesion.^1^ We found only two case reports of multiple tracheobronchial lesions, with tracheobronchial spread occurring seven and eight years after surgical treatment, respectively.^1^ ^,^ ^2^ In the case reported here, tracheobronchial spread occurred nine years after the second surgical procedure and 12 years after the first. 
